# Resilience, stress, and coping among Canadian medical students

**Published:** 2014-12-17

**Authors:** Behruz Rahimi, Marilyn Baetz, Rudy Bowen, Lloyd Balbuena

**Affiliations:** 1 Department of Internal Medicine, University of Alberta; 2 Department of Psychiatry, University of Saskatchewan

## Abstract

**Background:**

Numerous studies have established that medical school is a stressful place but coping styles and resilience have not been adequately addressed as protective factors.

**Method:**

Using a cross-sectional design, 155 students were surveyed using the Connor-Davidson Resilience Scale, Perceived Stress Scale, and the Canadian Community Health Survey Coping Scale. Mean scores were compared by gender and between our sample and normative scores using t-tests. Multivariate linear regression was performed to examine whether stress levels were related to coping and resilience.

**Results:**

Medical students had higher perceived stress, negative coping, and lower resilience than age and gender-matched peers in the general population. Male medical students had higher positive coping scores than general population peers and higher resilience, and lower perceived stress than female medical students. Coping scores did not vary by gender in our sample. The multivariate model showed that resilience and negative, but not positive coping, predicted stress.

**Conclusions:**

Medical students are neither more resilient nor better equipped with coping skills than peers in the population. Greater emphasis on self-care among medical trainees is recommended. Emphasizing the importance of self-care during medical training, whether by formal incorporation into the curriculum or informal mentorship, deserves further study.

## Introduction

Training to become a physician—a vocation dedicated to patient care—can be detrimental to one’s health. Stress, which can be defined as an unpleasant feeling of strain resulting from external demands,^1^ comes in different forms during medical training. The demands include heavy academic workloads,^2^ sleep disruption,^3^ on-call schedules, and exposure to life-and-death situations.^4^ The transitions involved in the various stages of training are particularly stressful.^5^ Consequently, mental health and life satisfaction typically worsen in medical school.^6^,^7^ US and Canadian medical students have higher levels of distress compared to the general population, and among students, women are more distressed.^8^ About half of medical trainees experience symptoms of burnout and 10 percent experience suicidal ideation to some extent.^9^,^10^ Student mental health challenges potentially affect patient care, with personal distress likely to decrease medical exam scores^9^ and empathy for patients.^11^ Some personality traits contributing to success in medical school—achievement drive and perfectionism—are also risk factors for impairment in professional practice.^12^

In response to this situation, several adaptive traits and trainee characteristics have been studied. Resilience—the ability to rebound from a stressful experience—has been suggested as a protective factor against adversity.^13^,^14^ Resilient individuals are able to function in the face of painful feelings, failure, and illness.^14^ No psychometric instrument is accepted as a gold standard measure of resilience.^15^ Its presence is instead inferred from the ability to withstand trauma and adverse events. A longitudinal study of medical students in Norway found that resilient students were characterized by stable levels of life satisfaction.^6^ A consistently high level of life satisfaction was in turn predicted by adequate social and leisure pursuits. A large sample study (n = 2,000) of Chinese medical students reported that resilience buffered negative life events while social support protected against mental health problems.^16^ The latter study found a gender difference, with males being more resilient than females.

Coping refers to a deliberate and planned process involving thoughts and actions intended to manage specific stressful demands.^17^ Coping can alter a stressful situation in two ways. First, it can be used to change the situation causing the stress. Strategies including information seeking, planning, problem solving, and developing new skills may be used for this purpose. Second, it can be used to manage the emotion itself and may include strategies such as mental and behavioural withdrawal, denial, distraction, distancing, reframing the problem and relaxation. According to the authors of the Norwegian study of medical student stress,^6^ coping styles were more important predictors of satisfaction than personality traits. The use of psychoactive substances may be either started or continued in medical school,^18^–^20^ although adaptive strategies such as positive reframing, seeking emotional support, engaging in leisure activities, religion, and planning are also utilized.^21^,^22^ Social contact with friends and family, and hedonic moments (having fun and laughing) help offset feelings of isolation.^23^

In an attempt to understand how medical students experience and cope with stress, we had two objectives in this study. First, we examined whether levels of stress, resilience, and coping in our sample of medical students were different from their counterparts in the general population. Second, we attempted to quantify the relative contributions of resilience, positive, and negative coping mechanisms to perceived stress.

## Method

### Sample

Our sample consisted of 155 first, second, third, and fourth year medical undergraduate students at the University of Saskatchewan. Prospective participants were invited to participate in an online survey entitled *Resilience and Coping Factors Related to Stress*. See Table 1 for sample characteristics. The study received approval from the Behavioural Research Ethics Board of the University of Saskatchewan.

### Instruments

Our instruments were three psychometrically validated scales measuring stress, resilience, and coping techniques. Resilience was assessed using the 10-item Connor-Davidson Resilience Scale (CD-RISC) scale.^14^ Items are rated from 1 (*not true at all*) to 4 (*true nearly all the time*). CD-RISC was been reported to have satisfactory reliability (Cronbach’s alpha = 0.85) and construct validity, and is among the three instruments with the best psychometric properties. 14,15 Higher CD-RISC scores represent greater tolerance for adversity. The 10-item Perceived Stress Scale (PSS), a widely used measure of stress^24^ was also used. The respondent is assessed with respect to the perception of life as being uncontrollable, unpredictable, and overloading.^25^ Items are rated from 0 (*never*) to 4 (*very often*). The PSS was reported to have a reliability of 0.89 in college students and good construct validity, correlating well with the State-Trait Anxiety Inventory—Trait scores.^26^ A coping scale used in the 2002 Canadian Community Health Survey (CCHS)—Mental Health and Well-being^27^ served as the measure of coping. The CCHS is an annual cross-sectional survey administered by Statistics Canada to Canadians 15 years or older and whose main objective is to collect health related data. The survey in 2002 included a module that focused on mental health and its determinants. The items in the CCHS Coping scale were derived from several coping scales and they assess the frequency with which a particular technique (e.g., jogging, drinking, sleeping) is used. Items are rated from 1 (*often*) to 4 (*never*). We reverse coded the original scale for ease of interpretation. Based on previous factor analytic work, the CCHS Coping scale was subdivided into positive and negative coping styles.^28^,^29^ Higher positive and negative coping scores indicate higher use of these particular techniques to cope with stress.

We calculated the mean negative and positive coping scores from the publicly available CCHS 1.2 dataset to serve as a benchmark for the scores in our sample. Since the CCHS surveyed the Canadian general population, we selected a subgroup of participants that matched our sample in age and gender.

### Study Design

Using a cross-sectional design, the survey was administered in the Fall and Winter terms of the 2011 school year through *SurveyMonkey.*^30^ *SurveyMonkey* is a proprietary software that facilitates online data collection and management. The survey invitation was sent to all 333 medical undergraduates and a total of 155 (47%) students provided informed consent. We made sure that surveys were at least two weeks removed from final examinations. Response rates by year level were 67%, 56%, 29%, and 34% from first to fourth year. There were 102 and 53 respondents in the Fall and Winter terms, respectively. In answering the survey, the participants used a unique numeric code, allowing us to link Fall and Winter responses. There were no changes with time in levels of resilience, coping, and stress so we pooled data from both periods. When the participant responded in both terms, we retained only the Fall response to maintain our cross-sectional design.

### Analysis

Within our sample we compared mean levels of stress, resilience, and coping scores by gender. We then compared medical student scores with normative scores for the PSS and CD-RISC in American adults.^25^,^31^ Negative and positive coping scores were compared with mean scores in the Canadian population. To keep the error rate of the multiple comparisons at 0.05, the alpha for each individual t-test was set at 0.005. All t-tests were two-tailed.

Univariate linear regression modeling, stratified by gender, was performed with stress as the dependent variable and resilience and positive/negative coping as predictors. Finally, multivariate regression modeling was performed to examine the net effect of resilience and coping styles on perceived stress. Statistical analyses were implemented in Stata.

## Results

Compared with the general population, female medical students had higher perceived stress (t = 6.21, df = 92, *p* < 0.001), negative coping (t =5.74, df = 89, *p* < 0.001), and lower resilience (t = −4.87, df = 92, *p* < 0.001). See Table 2 for details. Female positive coping scores were not different from their general population counterparts (*p* < 0.10). Males followed the same pattern as females, with higher perceived stress (t = 3.01, df = 54, *p* < 0.001), negative coping (t = 2.77, df = 51, *p* < 0.001), and lower resilience (t = −3.23, df = 54, *p* < 0.001) when compared with the general population. Males had higher positive coping against their general population peers (t = 1.98, df = 51, *p* < 0.05), but this difference did not survive significance after Bonferroni-adjustment.

Within our sample, male students had statistically higher resilience scores compared to females (t = 2.72, df = 146, *p* < 0.001). *See* Table 2 for details. Perceived stress (t = 2.72, df = 146, *p* < 0.001) was higher in females compared to males. Positive (*p* = 0.04) and negative (*p* = 0.07) coping scores did not vary by gender.

Our univariate models showed that negative coping varies directly (beta = 0.99 for females; 1.11 for males) with stress while resilience varies inversely (beta = −0.64 for females; −0.58 for males). See Table 3 for details. While positive coping was inversely related to perceived stress in women (beta = −0.56), no such relationship was found in men. Multivariate regression indicated that only resilience (beta = −0.45 for females; −0.32 for males) and negative coping (beta = 0.76 for females; 0.95 for males) are independently related to stress. With resilience and negative coping already entered in the model, positive coping was no longer significant

## Discussion

Our study has two major findings. First, medical students have higher levels of stress but are not more resilient than a sub-set of the population matched by age and gender. Second, among medical students, there is a gender difference in perceived stress, resilience, and coping.

The finding of higher levels of perceived stress among our medical students *vis-a-vis* the general population is consistent with previous studies.^32^,^33^ These two main findings reveal the irony that medical competence is, for many, acquired at some cost to one’s health. Though some stressors, such as the exposure to life-and-death situations and academic pressure, may be inevitable, this does not imply that methods cannot be devised to reduce the stress or that the system itself cannot be modified. The practice of medicine is governed by the Hippocratic principle of doing no harm to patients. Perhaps the principle should be applied also in the training of would-be-physicians so that they are both better able to handle necessary stressors and less exposed to unnecessary ones. This is especially important in light of the finding that student distress causes depersonalization and reduces empathy.^10^,^11^,^34^ It would be ideal that students leave medical school with the sense of compassion, empathy, and altruism that they had upon entering medical school.^35^ We believe that medical education should not only endow competence but preserve one’s “soul.”

The finding that medical students are less resilient than the general population, if true, is a potential cause for concern. This finding needs to be confirmed in additional studies because it is possible that lower resilience among our sample simply reflects higher levels of perceived stress. To our knowledge, ours is the first study to compare resilience levels of medical students with a population norm. A study of Chinese medical students reported mean scores that were lower than those of university students from other countries.^16^ They did not however compare the scores with the general population in China though a quick glance at the reported mean CD RISC scores of Chinese medical students shows they are well below US general population norms.^36^ If these findings are real the courses of action that medical schools take to improve student resilience may depend on how this concept is conceptualized. If resilience is considered to be predominantly a preformed and stable part of personality upon entry to medical school, then it would be important for medical schools to admit candidates who have the psychological resources to cope with medical school challenges. If on the other hand, resilience is thought to be a modifiable characteristic, perhaps like a reservoir that fluctuates depending on demand and replenishment, 37 then assisting students once they enter medical school matters. One recent commentary supports training students in resilience instead of eliminating or not admitting candidates with low resilience, because medical schools would not wish to unintentionally screen out dedicated, empathetic individuals who have not acquired sufficient resilience.^38^ Howe and colleagues have outlined actions that medical schools can take in order to develop the resilience of students.^39^ Our own data suggest that medical schools could do more to support the use of active, positive coping mechanisms and discourage avoidant, escapist, and other negative coping strategies.

The finding that female medical students have higher levels of perceived stress than males has been reported previously but the findings have not been consistent. Two Pakistani studies 2,21 and a Swedish study 32 reported higher levels of stress among females as compared with males. On the contrary, a Finnish 40 and a British^33^ study did not find gender differences in stress. In our sample, the pattern of differences in stress and resilience seemed less favorable for female students. Our multivariate model showed that, for female students, after accounting for resilience and negative coping, positive coping was no longer inversely related to stress. Further research needs to be done to help determine whether certain aspects of medical education need to be more attuned to gender differences.

Of the three variables we examined, coping is perhaps the most modifiable and subject to behavioural intervention. We found that male medical students have higher positive coping skills than their age-matched peers in the general population. This finding needs to be confirmed, but if true, positive coping among all students should be nurtured. Incorporating self-care and balanced living as part of the curriculum might help first year students learn coping skills that can be applied throughout medical school. Medical students at a Chicago medical school were required to undergo a six-week healthy living course^41^ targeting exercise, nutrition, sleep, personal habits, work/study habits, and mental emotional health. The great majority of students reported the program as valuable. A Norwegian study likewise recommended that medical schools encourage students to pursue social and leisure activities to promote health.^6^ There is a need to study whether formalizing health promotion as a course requirement or leaving it to the students’ initiative is more effective.

Our project is subject to some important limitations. First, except for the CCHS Coping Scale, the norms used for perceived stress and resilience are those for an American population. Hence, it is possible that compared to Canadian counterparts, medical students might not have the same levels of perceived stress. Secondly, the CCHS Coping scale, our measure of negative and positive coping, has Cronbach alphas of .59 and .37, respectively for the subscales which fall below the acceptable range. Although this seems like a serious issue, one would not expect a list of positive coping styles to have high correlations among the items since the use of a technique such as exercise would not necessarily be related to another one such as meditation. We used the CCHS Coping scale because it allows for a comparison of student scores with age and gender matched individuals in the population. In our assessment, this benefit outweighed the instrument’s limitations. Third, although we measured levels of stress, we did not have information about the particular stressors experienced.

## Conclusion

Despite these limitations, our findings contribute to the growing evidence of high levels of stress and poor coping and resilience among physicians and medical students. Ways of responding to the stress associated with medical training—such as the capacity to elicit support from peers, interactions with mentors, and habits of balanced living are all acquired in medical school and perhaps stay with physicians throughout their careers. We might anticipate that not only the medical students but also their families, friends, and future patients, might benefit from positive health investments and changes made in medical school.

## Figures and Tables

**Table 1:** Demographic characteristics of medical students

Characteristic	Frequency (%)
N (sample size)	155
Gender	
Male	56 (36)
Female	98 (64)
Mean age (SD)	24.15 (3.88)
Year in Medicine	
I	56 (36.13)
II	47 (30.32)
III	24 (15.48)
IV	28 (18.06)
Civil status	
Single	115 (74.19)
Married/common-law	38 (24.52)
Missing	2 (1.29)

**Table 2:** Resilience, stress, and negative coping by gender

	Within sample comparisons			Sample vs norm comparisons	
Measure	n	Sample Mean (SD)	P	Norm Mean (SD)	p
CD-RISC (Resilience)					
*Female*	93	28.84 (4.44)	< 0.001	31.08 (5.56)	< 0.001
*Male*	56	31.25 (5.23)		33.53 (4.60)	< 0.01
Perceived Stress Scale					
*Female*	93	17.41 (5.76)	< 0.001	13.7 (6.6)	< 0.001
*Male*	55	14.65 (6.29)		12.1 (5.9)	< 0.001
Negative Coping Index*					
*Female*	90	16.59 (2.94)	NS	14.76 (3.30)	<0.001
*Male*	52	15.58 (3.59)		14.03 (3.37)	<0.001
Positive Coping Index*					
*Female*	90	19.09 (2.43)	NS	18.67 (2.62)	NS
*Male*	52	18.23 (2.15)		17.64 (2.76)	NS

Note: Scores in the CD-RISC range from 10 to 40 and are in higher is better form. Scores in the Perceived Stress Scale range from 0 to 40 and are in lower is better form. Scores in the Positive Coping Index range from 6 to 24 and in the Negative Coping Index from 7 to 28. Higher scores in these Coping Scales represent more frequent use of techniques listed in the 2002 CCHS. The normative scores for each scale are from references 24, 26, and 30.

**Table 3:** Univariate and multivariate regression models of perceived stress

	Univariate models			
	Females		Males	
	Beta (95% CI)	*p*	Beta (95% CI)	*p*
*Model 1:* Resilience	−0.64 (−.88 to −.41)	< 0.01	−0.58 (−0.87 to −0.29)	< 0.01
*Model 2:* Negative Coping	0.99 (0.63 to 1.34)	< 0.01	1.11 (0.72 to 1.50)	< 0.01
*Model 3:* Positive Coping	−0.56 (−1.04 to −0.07)	0.03	−0.62 (−1.43 to 0.19)	0.13
	**Multivariate models**			
*Model 4:*	Beta (95% CI)	*p*	Beta (95% CI)	*P*
Resilience	−0.45 (−.70 to −.20)	< 0.01	−0.32 (−.61 to −.03)	0.03
Negative Coping	0.76 (0.39 to 1.12)	< 0.01	0.95 (0.57 to 1.34)	< 0.01
Positive Coping	−0.01 (−0.45 to 0.43)	0.98	−0.20 (−0.87 to 0.47)	0.55
